# The Victorian Sports Assessment Institute-Achilles Tendinopathy Questionnaire (VISA-A): Chinese cross-cultural adaptation and psychometric validation

**DOI:** 10.1186/s12955-022-02025-6

**Published:** 2022-07-23

**Authors:** Xiaoxian Tu, Zhiyuan Tu, Wei Lin, Zhe Wu

**Affiliations:** 1 Medical Records Management Room, Fujian Medical University Union Hospital, Fuzhou, China; 2 Orthopedics, Fujian Provincial Corps Hospital of Chinese People’s Armed Police Force, Fuzhou, China; 3 Physical Education Institute of Jimei University, Xiamen, China

**Keywords:** Translation, Reliability, Validity, Chinese, Cross-cultural adaptation, The VISA-A, Achilles tendinopathy

## Abstract

**Purpose:**

We aimed to create a standardized cross-cultural adaptation of the simplified Chinese version of VISA-A, test its reliability and validity and conduct exploratory factor analysis on the correlation between items.

**Methods:**

According to international recommendations for the cross-cultural adaptation of questionnaires, after considering the opinions of patients, we translated and revised the English version to create a simplified Chinese version of the questionnaire. We recruited healthy subjects in the general specialty of one university (n = 90) and the physical education specialty of another university (n = 89), and we recruited patients with Achilles tendinopathy in a third group (n = 85). Reliability was evaluated by calculating test–retest reliability and internal consistency, validity was evaluated by exploring structural and criterion validity (correlation with the physical function and body pain items of the SF-36), and responsiveness was evaluated by calculating area under the receiver operating characteristic curve (AUC).

**Results:**

The simplified Chinese version of the VISA-A had no ceiling or floor effects. Four common factors were extracted and explained by the exploratory factor analysis. The test–retest reliability (ICC = 0.97) and internal consistency (Cronbach’s alpha = 0.84) were adequate. The questionnaire had moderate correlations with the physical function and body pain items of the SF-36. The AUC was 0.9407.

**Conclusion:**

The simplified Chinese version of the VISA-A had good reliability and validity and excellent responsiveness, but the factorial structure is not inconsistent with the dimensions of the original version. It can be used to assess and manage patients with Achilles tendinitis in the Chinese culture.

## Introduction

Achilles tendinopathy is generally regarded as a clinical syndrome of pain, swelling, and dysfunction caused by the overuse of the ankle. The Achilles tendon is the largest and strongest tendon in the human body, but it is also one of the tendons prone to degeneration and injury. Histopathological study of Achilles tendinopathy tissue has not found inflammatory cells but instead found that the repair of Achilles tendon tissue damage can fail, and degenerative lesions can occur. Repeated stress, such as running and jumping, is considered a common cause of Achilles tendon injuries [1, 2].

The proportion of soldiers and athletes who come to our hospital for treatment is high, and Achilles tendinopathy is common. We have been exploring quantifiable indicators of Achilles tendinopathy to guide clinical treatment. The Victorian Sports Assessment Institute-Achilles Tendinopathy Questionnaire (VISA-A) was developed in 2001 by the Victoria Sports Research Group to measure the severity of Achilles tendinopathy [3]. The VISA-A is a patient self-rating scale for Achilles tendinitis, consisting of 8 questions regarding pain and function and the consequences of participating in sports [4]. The VISA-A was developed for English-speaking people, but it has been translated into French, Swedish, Italian, German, Turkish, and Dutch, and its reliability and validity have been tested [5–10]. With the development of the national fitness movement in China, there has been an increase in patients with Achilles tendinopathy. Therefore, this study was performed. After the cultural adaptation of the Chinese translation version of the VISA-A, its reliability, validity, and responsiveness were tested in three groups of ordinary college students, sports college students, and patients with Achilles tendinopathy.

## Methods

### Cross-cultural adaptation

We contacted author J L Cook of the original English version of the VISA-A, and he agreed to our use. We referred to the International Recommendations for Intercultural Adaptation of Questionnaires for Measuring Health Status by Beaton et al. [11, 12]. For linguistic and cultural equivalence, the questionnaire underwent six translation and cultural adaptation steps to be applied to Chinese-speaking patients.

Step 1: Initial translation

The questionnaire was translated from English to Chinese. There were two translators, both of whom are native speakers of Chinese; one has medical knowledge and is engaged in clinical work, while the other translator is outside the medical field and works in physical education. After independent translation, both translators provided written Chinese versions of the questionnaire (V1.1, V1.2).

Step 2: Merging of the translations

The two translators collaborated to merge the two initial translations into a new Chinese scale (V2.0).

Step 3: Back-translation

The Chinese scale was then back-translated into English. The combined version of the questionnaire was translated back to its original language, English (VB1), by another native English-speaking bilingual individual who had not been exposed to the overdose form and who works outside the medical field.

Step 4: Expert committee

A special expert committee consisting of an orthopedic specialist, a linguist, and those who initially participated in the translation was formed. The committee reviewed all translated versions and produced a test version (V3.0).

Step 5: Pilot testing of “V3.0 version”

Ten healthy people and 10 patients with Achilles tendinopathy were recruited for testing. After filling out the questionnaire, subjects reported on their understanding of the different items and the answers they provided, the statements they did not understand, and their suggested revisions, and they discussed the difficulty of answering questions. After the suggestions were collected, they were reviewed and revised by the expert committee to form a new test version (V4.0).

Step 6: Pilot testing of V4.0 version

Ten healthy people and 10 patients with Achilles tendinopathy were recruited again for testing. The subjects reported on their understanding of the test items and the answers they provided, and their suggestions were collected, reviewed by the expert committee, and revised to form a new version (V5.0).

Step 7: Approval by the expert committee

Having determined that there was no misunderstanding or confusion about any of the items, the expert committee approved the final version of the VISA-AC questionnaire.

After translation and cross-cultural adaptation, we validated the VISA-AC questionnaire.

### Study population

This study is part of a clinical trial that has been approved by our hospital. For exploratory factor analysis, 10 times the number of entries was required, and all groups recruited at least 80 volunteers [13]. From August 2020 to October 2021, three groups of subjects were recruited: group 1 included college students who were not majoring in sports; group 2 included college students majoring in sports; and group 3 included outpatients in our hospital with unilateral Achilles tendinopathy who were diagnosed by two orthopedic doctors. The patients included active-duty soldiers, athletes, and ordinary professionals. All participants were over 18 years old, all had the right to provide informed consent, and all understood the purpose and significance of this study. Pregnant women, lactating women, patients with bilateral Achilles tendinopathy, and those with other diseases or trauma affecting function and exercise levels were excluded.

Participants in groups 1 and 2 completed the questionnaire anonymously twice: at study enrollment (time 0) and 1 week after initial testing (time + 7 days). The third group completed the VISA-A questionnaire and the physical function (PF) and bodily pain (BP) items from the SF-36 on the first visit. One hundred questionnaires were distributed to groups 1 and 2; 90 qualified questionnaires were collected in the first group, and 89 eligible questionnaires were collected in the second group. A total of 85 suitable questionnaires were collected from the third group within the planned time. There were three groups of investigators who were trained and supervised by the same individual from the expert committee.

### Data management and statistical analysis

Data were analyzed using SAS OnDemand for Academics (SAS INSTITUTE, INC.). The significance level was 0.05, and the statistical power was 0.8. The percentages of subjects with 0 and 100 points on the VISA-AC were calculated to show any floor and ceiling effects in the tendinopathy group. Factor analysis was carried out by principal component analysis and the maximum variance rotation method, combined with clinical significance, and the factors with eigenvalues ​​greater than 1 or the number of factors with a cumulative contribution rate of 70% were retained. Internal consistency was calculated using Cronbach’s alpha for the tendinopathy group. A Cronbach's alpha of 0.7–0.9 demonstrates acceptable internal consistency. The test–retest reliability was calculated for the first and second groups using absolute agreement with a two-way mixed-effects model of intraclass correlation coefficient (ICC). ICC ≥ 0.7 is considered acceptable [14]. The VISA-A scores for the first measurement of the three groups were compared using one-way ANOVA and then between each group using Scheffe's post-hoc test based on histograms, provided that the data showed an approximately normal distribution. The area under the receiver operating characteristic curve is an indicator for judging the classifier's quality, and we used it to evaluate the scale's responsiveness. The scores in the tendinopathy group and the scores for the PF and BP items from the SF-36 were subjected to correlation analysis. They are predicted to have moderate to strong correlations based on other versions' results. Weak correlations are less than 0.3, moderate correlations between 0.3 and 0.6, and strong correlations above 0.6 [14]. We performed a post hoc sensitivity analysis of nonparametric tests on the parametric test results to test their robustness.

## Results

The characteristics of the subjects are shown in Table 1. There were 90 general professional college students, including 47 males and 43 females, with an average age of 20.4 ± 0.9 years and an average body mass index (BMI) of 20.4 ± 3.1 kg/m^2^. Eighty-nine college students were majoring in physical education, including 50 males and 39 females, with an average age of 20.9 ± 0.9 years and an average BMI of 23.0 ± 3.6 kg/m^2^. The tendinopathy group consisted of 85 patients, 51 males, and 34 females, with unilateral onset; the average age was 27.2 ± 10.1 years, and the average BMI was 21.0 ± 2.0 kg/m^2^. Weekly exercise time was calculated in hours, and the average values for three groups were 5.2 ± 3.5 h/week, 10.9 ± 4.4 h/week, and 19.4 ± 9.5 h/week.

**Table 1:** The characteristics of the subjects

	General students (n = 90)	Sports students (n = 89)	Achilles tendinopathy (n = 85)
Age	20.4 ± 0.9	20.9 ± 0.9	27.2 ± 10.1
*Gender*			
Men	47	50	51
Women	43	39	34
Body Mass Index	20.4 ± 3.1	23.0 ± 3.6	21.0 ± 2.0
Hours training/week	5.2 ± 3.5	10.9 ± 4.4	19.4 ± 9.5

During the translation and cultural adaptation stage, the expert committee discussed and resolved some disputes in the translation and made some adjustments according to the subjects' opinions without changing the expressions of the original English version. We changed the term "box" in items 1–6 of the original version to "scale" so that subjects could better understand the meaning of the score after the change (S1).

Ceiling or floor effects are said to be present if more than 15% of respondents scored the maximum or minimum possible score [15]. The scores of patients with Achilles tendinopathy were counted, with no subjects with scores of 100 or 0 subjects and thus no ceiling and floor effects.

For the exploratory factor analysis, Bartlett’s test of sphericity was significant (*P* < 0.0001) and the Kaiser–Maeyer–Olkin (KMO) was 0.82 indicating suitability for the factorial analysis. The rule determines the number of factors for which the eigenvalue is greater than 1 or the sum of the largest eigenvalues ​​accounts for more than 70% of the total eigenvalues. Therefore, the first four latent factors with a cumulative contribution rate of 77.9% were taken. The relationship between latent factors and indicators was determined according to the principle of variance exceeding 50%. Factor 1 includes Item 5, Item 6, and Item 7; Factor 2 includes Item 2 and Item 4; Factor 3 includes Item 3 and Item 8; and Factor 4 includes Item 1. Rotated Factor Pattern are in Table 2.

**Table 2:** Rotated factor pattern

	Factor 1	Factor 2	Factor 3	Factor 4
Item 1	0.13847	0.23759	0.19524	0.90204
Item 2	0.00315	0.78841	0.24369	0.18969
Item 3	0.04220	0.26234	0.88224	0.11318
Item 4	0.42712	0.70296	0.10469	0.30721
Item 5	0.86002	− 0.02019	0.01125	0.21988
Item 6	0.71609	0.43085	0.21603	− 0.00013
Item 7	0.55878	0.50502	0.29162	− 0.09420
Item 8	0.49913	0.12442	0.62819	0.30018

Internal consistency using Cronbach's alpha calculated separately for the three groups were 0.82, 0.81, and 0.84. Because the patients with Achilles tendinopathy were treated after being seen in the outpatient clinic, only general college students and sports primary college students were evaluated for test–retest reliability. Both groups scored 0.97 (0.95–0.98), and the effect was good. The psychometric properties of the VISA-AC are summarized in Table 3.

**Table 3:** Psychometric properties of the VISA-AC

Measurement property	Result
*Cronbach’s alpha*	
General students (n = 90)	0.82
Sports students (n = 89)	0.81
Achilles tendinopathy (n = 85)	0.84
*ICC (95% CI)*	
General students (n = 90)	0.97 (0.95–0.98)
Sports students (n = 89)	0.97 (0.95–0.98)
*Spearman’s rho (SF-36) (n* = *85)*	
VISA-VC VS PF	0.53, *P* < 0.0001
VISA-VC VS BP	0.61, *P* < 0.0001
*Ceiling and floor effect (n* = *85)*	
% of patients with the maximum score	0%
% of patients with the minimum score	0%

PF, physical function; BP, bodily pain

The scores of the three groups were approximately normally distributed. The differences among the three groups were statistically significant, with *P* < 0.0001. Using the Scheffe post hoc test, the results showed that the scores of the ordinary college students and sports college students were lower than those of the Achilles tendinopathy group, and the difference was statistically significant, *P* < 0.0001. The difference in scores between ordinary college students and sports college students was − 4.5 (95% CI − 9.4 to 0.4), *P* = 0.08, the difference was not statistically significant. However, the P value was close to 0.05, so a t test was performed between the two groups on the 8th item with the highest score, and the result of difference in score was − 3.1 (95% CI − 4.9 to − 1.3), *P* = 0.0008. The psychometric properties of the VISA-AC are summarized in Table 4. The nonparametric sensitivity analysis was similar to the parametric analysis results for the above results (S2).

**Table 4:** The one-way analysis of variance to the three groups and the post hoc test

Measurement property		Mean ± SD	Result
*ANOVA*			
General students (n = 90)		90.5 ± 11.1	*P* < 0.0001
Sports students (n = 89)		95.0 ± 7.8	
Achilles tendinopathy (n = 85)		60.3 ± 18.9	
*Scheffe's post-hoc test*			
General students (n = 90) comparison Sports students (n = 89)			*P* = 0.08, − 4.5 (− 9.4 to 0.4)
General students (n = 90) comparison Achilles tendinopathy (n = 85)			*P* < 0.0001, 30.2 (25.2 to 35.1)
General students (n = 89) comparison Achilles tendinopathy (n = 85)			*P* < 0.0001, 34.7 (29.7 to 39.7)
*Item 8* *t* test			
General students (n = 90)		24.8 ± 7.2	*P* = 0.0008, − 3.1 (− 4.9 to − 1.3)
Sports students (n = 89)		27.9 ± 4.9	

The correlation between the PF and BP items in different language versions of the VISA-A and SF-36 has been verified. The Chinese version of the SF-36 is widely used, so we performed Spearman's rho correlation analysis on the VISA-AC score and PF and BP scores of patients with Achilles tendinopathy for criterion validity. The results showed a correlation between the VISA-AC and PF scores, rs = 0.53, *P* < 0.0001, and there was also a correlation between the VISA-AC and BP scores, rs = 0.63, *P* < 0.0001.

**Fig. 1:**
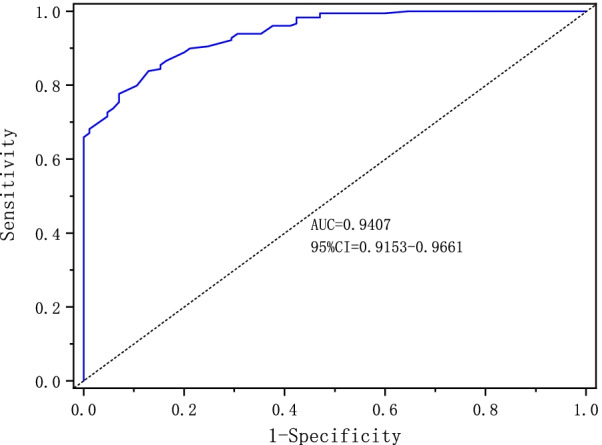
The area under the receiver operating characteristic curve, indicating that the simplified Chinese version of VISA-A can distinguish between healthy people and patients with Achilles tendinopathy. The area under the curve (AUC) is 0.9407, and the 95% confidence interval is 0.9153–0.9661

The area under the receiver operating characteristic curve (AUC) was calculated from the first VISA-A scores of healthy people and patients with Achilles tendinopathy, with an AUC = 0.9407 (95% CI 0.9153–0.9661), which was larger than 0.9. Therefore, the scale had an excellent ability to measure the characteristics of different objects (Fig. 1).

## Discussion

The VISA-A has been translated into many languages, including French and Spanish, with a significant population. At the time our research was initiated, there was no Chinese version of the VISA-A, but during our literature update phase, we found that a simplified Chinese version of the study had been published [16]. However, there are some differences between the study population used to create this version and our study population. The two studies were independent, with different subjects, indicating that the VISA-A has received increasing attention from doctors and is widely used in the self-management and assessment of Achilles tendinopathy.

During the cultural adaptation, some Chinese language adjustments were made upon the subjects’ suggestion, but these adjustments did not change the meaning of the original English version. For the scoring instructions in questions 1–6, we first followed the original version and used the term "boxes" to describe the score options, but some subjects did not understand the meaning; this problem did not occur again after we used the revised version of the scale. For the translation of question 7, to make it understandable to healthy patients, we translated "when symptoms began" to "when symptoms began (or before)." Other parts were left unchanged during translation and cultural adaptation.

For the healthy subjects in this study, we chose college students, but we selected two types of college students: ordinary college students and college students majoring in sports. The Achilles tendinopathy patients in this study included military athletes and general professionals [17]. We counted the weekly exercise time of the three groups of subjects, which was calculated in "hours/week," which is more accurate than "days/week." Judging from the average weekly exercise time of the three groups, the patients with Achilles tendinopathy exercised longer than the healthy subjects. Among the healthy subjects, the time spent on exercise of sports majors was higher than that of ordinary college students.

The finding that the simplified Chinese version of the VISA-A had no floor and ceiling effects suggests excellent discriminating ability of the questionnaire.After we performed exploratory factor analysis with the VISA-AC, there were two-factor eigenvalues ​​greater than 1, but the cumulative contribution rate was only 60.30%. After rotation, factor 1 included Item 1–Item 4, and factor 2 included Item 5–Item 8. Based on the meaning of the entries, we could not name these two factors. Based on the principle that the cumulative contribution rate should exceed 70%, we selected 4 factors to retain. According to clinical significance, factor 1 including Item 5, Item 6, and Item 7 was associated with exercise level factors, factor 2 including Item 2 and Item 4 was correlated with Achilles tendon stretching, and factor 3 including Item 3 and Item 8 was associated with exercise level. The time factor was related, and the factor including Item 1 was related to morning pain. According to the current research on exploratory aspects, the Swedish and Persian versions [10, 18], our version, and another Chinese version all have two factors whose eigenvalues ​​are more significant than 1 [16]. Except for our study, other studies extracted two elements, "pain and symptoms" and "physical activity," but their factor loading items were different. Spanish researchers performed confirmatory factor analysis and suggested that the VISA-A is a univariate scale [6]. These studies all yielded different designs than the original design of the original English version, which had three dimensions. We do not think this is a problem with other versions that have been translated and undergone cross-cultural adaptation but rather that it is a problem with the original scale design. The structural issues with the original version were previously questioned in an editorial [19].

A previous meta-analysis suggested that Cronbach's alpha should be higher than 0.75 and that an ICC of 0.91 is acceptable [20]. The Cronbach's alpha coefficients of all three groups in our study were higher than 0.8, so the internal consistency of our version of the VISA-AC was good. The subjects with Achilles tendinopathy were outpatients, and patients will only go to the doctor if they experience signs and symptoms that affect their lives. If they had not received treatment, they would have started the treatment only after the second questionnaire survey 1 week later, which could not be done due to reasons of ethics and patient acceptance. If the questionnaire survey had been conducted after 1 week of treatment, then the treatment factor could not have been removed, so we only tested the repeated validity of the two groups of college students. The ICCs of both groups were 0.97 (95% CI 0.95–0.98), and the test–retest reliability was good.

The time to complete the scale was kept within 5–7 min, and patient acceptance was better. For example, it takes a long time to complete the VISA-A and the SF-36 scales at one time, which affects the authenticity of the survey responses. Second, multiple language versions of the study have confirmed the correlation of the VISA-A with the PF and BP items of the SF-36 [16, 18, 20]. Therefore, we verified the validity of the two content items of the VISA-AC and the PF and BF items. Our results reconfirmed the correlation between the VISA-A and PF and BP items.

One-way analysis of variance showed differences in the scores among the three groups. The Scheffe post hoc test between each pair of groups found that the scores of the Achilles tendinopathy group were lower than those of the other two groups and that the differences between the groups were statistically significant. This finding explains why the scale has good discrimination between healthy patients and patients with Achilles tendinopathy. There was a difference between the scores of physical education college students and general professional college students, mean difference =  − 4.5. Considering the difference in exercise time between the two groups, we performed a post hoc statistical analysis of the between-group scores for the eighth item, and the results were different. Therefore, the VISA-AC has good discriminant validity.

The receiver operating characteristic curve is generally used to formulate diagnostic criteria in medicine, but the VISA-A is a self-management scale and has no diagnostic effect. However, the receiver operating characteristic curve is also a valuable tool for studying binary classification problems, considering both sensitivity and specificity. The scale needs responsiveness to distinguish between different objects and changes in target characteristics and reflect the sensitivity to changes in characteristic values. The area under the receiver operating characteristic curve has this property, and our calculation results verified that the VISA-AC has good responsiveness [14].

There are certain limitations of our research. Patients with Achilles tendinopathy were not surveyed a second time with the VISA-AC after 1 week. No studies were performed on the minimal clinically meaningful change. A longitudinal study is required to address these limitations.

## Conclusion

The simplified Chinese version of the VISA-A was successfully adapted and had good reliability and validity and excellent responsiveness. However, the factorial structure is not inconsistent with the dimensions of the original version. The questionnaire can be used to assess and manage patients with Achilles tendinitis in the Chinese culture.

## Data Availability

Not applicable.

## References

[1] Longo UG, Ronga M, Maffulli N (2018). Achilles tendinopathy. Sports Med Arthrosc Rev.

[2] Liu CJ, Yu KL, Bai JB, Tian DH, Liu GL (2019). Platelet-rich plasma injection for the treatment of chronic Achilles tendinopathy: a meta-analysis. Medicine (Baltimore).

[3] Robinson JM, Cook JL, Purdam C (2001). The VISA-A questionnaire: a valid and reliable index of the clinical severity of Achilles tendinopathy. Br J Sports Med.

[4] Zhang YJ, Xu SZ, Gu PC (2018). Is platelet-rich plasma injection effective for chronic achilles tendinopathy? A meta-analysis. Clin Orthop Relat Res.

[5] Keller A, Wagner P, Izquierdo G (2018). Cross-cultural adaptation and validation of the VISA-A questionnaire for Chilean Spanish-speaking patients. J Orthop Surg Res.

[6] Hernández-Sánchez S, Poveda-Pagán EJ, Alakhdar-Mohmara Y, Hidalgo MD, Fernández-de-Las-Peñas C, Arias-Buría JL (2018). Cross-cultural Adaptation of the Victorian Institute of Sport Assessment-Achilles (VISA-A) questionnaire for Spanish athletes with achilles tendinopathy. J Orthop Sports Phys Ther.

[7] de Mesquita GN, de Oliveira MNM, Matoso AER, de MouraFilho AG, de Oliveira RR (2018). Cross-cultural adaptation and measurement properties of the Brazilian Portuguese version of the Victorian Institute of Sport Assessment-Achilles (VISA-A) questionnaire. J Orthop Sports Phys Ther.

[8] Kaux JF, Delvaux F, Oppong-Kyei J (2016). Validity and reliability of the French translation of the VISA-A questionnaire for Achilles tendinopathy. Disabil Rehabil.

[9] Iversen JV, Bartels EM, Jorgensen JE (2016). Danish VISA-A questionnaire with validation and reliability testing for Danish-speaking Achilles tendinopathy patients. Scand J Med Sci Sports.

[10] Silbernagel KG, Thomee R, Karlsson J (2005). Cross-cultural adaptation of the VISA-A questionnaire, an index of clinical severity for patients with Achilles tendinopathy, with reliability, validity and structure evaluations. BMC Musculoskelet Disord.

[11] Beaton DE, Bombardier C, Guillemin F, Ferraz MB (2000). Guidelines for the process of cross-cultural adaptation of self-report measures. Spine (Phila Pa 1976).

[12] Mokkink LB, Terwee CB, Patrick DL (2010). The COSMIN study reached international consensus on taxonomy, terminology, and definitions of measurement properties for health-related patient-reported outcomes. J Clin Epidemiol.

[13] Sousa VD, Rojjanasrirat W (2011). Translation, adaptation and validation of instruments or scales for use in cross-cultural health care research: a clear and user-friendly guideline. J Eval Clin Pract.

[14] Prinsen CAC, Mokkink LB, Bouter LM (2018). COSMIN guideline for systematic reviews of patient-reported outcome measures. Qual Life Res.

[15] McHorney CA, Tarlov AR (1995). Individual-patient monitoring in clinical practice: are available health status surveys adequate?. Qual Life Res.

[16] Chang R, Tsang RC, Jiang D (2021). Cross-cultural adaptation and measurement properties of the VISA-A questionnaire for Chinese patients with Achilles Tendinopathy. Phys Ther Sport.

[17] Rabin A, Kozol Z, Finestone AS (2014). Limited ankle dorsiflexion increases the risk for mid-portion Achilles tendinopathy in infantry recruits: a prospective cohort study. J Foot Ankle Res.

[18] Bahari M, Hadadi M, Vosoughi AR, KordiYoosefinejad A, Sobhani S (2020). Cross-cultural adaptation, reliability and validity of the Persian version of the Victorian Institute of Sport Assessment-Achilles questionnaire (VISA-A). Disabil Rehabil.

[19] Mallows A, Littlewood C, Malliaras P (2018). Measuring patient-reported outcomes (PROs/PROMs) in people with Achilles tendinopathy: how useful is the VISA-A?. Br J Sports Med.

[20] Palazon-Bru A, Tomas-Rodriguez MI, Mares-Garcia E, Gil-Guillen VF (2019). A reliability generalization meta-analysis of the Victorian Institute of Sport Assessment Scale for Achilles Tendinopathy (VISA-A). Foot Ankle Int.

